# Supplementary material for: Patterns of Intron Gain and Loss in Fungi
Source: PLoS Biol. 2004 Nov 30;2(12):e422. doi: 10.1371/journal.pbio.0020422 (PMC532390; doi:10.1371/journal.pbio.0020422)
Supplement: Table S1 — Also available at http://genes.mit.edu/NielsenEtAl/. (4.3 MB ZIP). [file pbio.0020422.st001.zip › NielsenEtAl/html/943.html]

AN6824.1.NCU03741.1.MG08159.1.FG07150.1


```
 CLUSTAL W (1.82) Multiple Sequence Alignments - Introns Inserted


Sequence 1: MG08159.1	639 aa
Sequence 2: FG07150.1	601 aa
Sequence 3: NCU03741.1	612 aa
Sequence 4: AN6824.1	548 aa
Alignment Length: 659 aa
Number Identitical Residues: 215 aa
Alignment Score (without introns) 10766


MG08159.1 	MGNSINNDKPLSPFAEKHISFDDG---HSSASDEDQKAVE~VQSRPSRSPRPEAPLQSAG
NCU03741.1	MA----------PDRERKPENDDH----SSSSEEDQDALE0VS-----------------
FG07150.1 	MG----------DHSEKHIHFQDGDYGSDGYSDDDGKAVE0HIQDTRKNGAANTEASSQV
AN6824.1  	MP----------SISSSPPR-------ADVIARSSFLALR~SAP----------------
          	*              .            .  : ..  *:.                    

MG08159.1 	ATLSCTSYRLEYLE~DQSRPLSGLLSIESTAAQYSSLHRA~EPK1SQRKLSEGSNQNS-Q
NCU03741.1	-FDPSSPYRRKSSL~VNSDARVPLHLRRSQPARTECLVHQ~FLD~AQRRPPRRQNTLDGQ
FG07150.1 	VFDPGNPYRRKSSM~VASEIKAPVMRHPNRRDE--CLVHQ~FLD~SQRRAKDAACSAS-D
AN6824.1  	--GPWKKHKRQNNA0PQPRTLTLVKGHDHKQENDPPKQFE0VLF~PRRTCSHRRKASLVM
          	   . . :: :      .     :        .            .:*            

MG08159.1 	DHGPHNAVTGDRQKERRKGR--SSPLSQALHPSVEQGGDKAVDPVGTSSRESSNGTANPG
NCU03741.1	VDGTASTSTNSIETRSPITRGLTDIARDAVQVSRENSQDQVLDPKGFSSTVG-NIAANVR
FG07150.1 	LDSETSMGNGSYISSRPDPS--QASLHKALNG----------DSLEAAHNEGLDSITDPK
AN6824.1  	ADEPRIDHTDEATACFVHSLIAGEGPMPAKIAN------------FRISKDRSKLEADIV
          	 .      ...                 *   .                    .  ::  

MG08159.1 	LKRRFDYLEAPSDSSDAGQVDEHHWRHRISKSQADIDMAGPSADLPEVLHSRLLTKKQLS
NCU03741.1	ARECSMTNANSSDTSDAGQVDEKEWKSEIVKAPN---------TDPEEKYSRLLTKKQLS
FG07150.1 	AKKKCRAVVEPGSMDHSGQADQQAWTQQMTKSMSEFDLG----SYQEDVRSRLLTKKQLS
AN6824.1  	GKSAAGAHDVAIEEEDEDEDDENQKPDAHGRAKS---------PSPDEVQSRHLTKKQLS
          	 :        . . .. .: *::       ::              :   ** *******

MG08159.1 	EMAWGVRELSRRLGSMRLKFRVKSIFLLTKIYDEDLIPKTRELTRWLLDKDRDVKYTVWV
NCU03741.1	EMAWGVRELSRRLSSVRLKFKIKTIFVLTKPYDQELIPKTRALIRWLLDKERDVRYTVYI
FG07150.1 	DMAWGVRELSRRLSSMRLRFKVKSIFILTKIYDQDLIPKTRELVKWLLNHNHEVAYTVYV
AN6824.1  	DMAWNVRNLSKKLDSIRLKLNVKSIFLVTKAGDESVVDATRQVARWLLSKDRGTQYVVYI
          	:***.**:**::*.*:**::.:*:**::**  *:.::  ** : :***.::: . *.*::

MG08159.1 	ERSLKENKKFDALGLLNELQHDYQSSGDPADEEGAKGVSRRLRYWDENICRTRPHTFDFV
NCU03741.1	DKALRCNKKLDAPGLIEEIRKDYVGSGEISEEAGND-ISERLRYWDEELCRARPQTADFV
FG07150.1 	QDKLKTNKKFDVSGIIDEVSKGYVQKDGANEQTVKETLSKRLRYWDENMCRTRPHTFDFV
AN6824.1  	ENKLRIDPEFDYESLVKEDSS----------------VGERLKFWDAKLASEQAHLFDLV
          	:  *: : ::*  .::.*                   :..**::** ::.  :.:  *:*

MG08159.1 	ITLGGDGTVLYASWLFQRIVPPVLSFALGSLGFLTKFDFDEHKDILTTAFNHGVTVSLRL
NCU03741.1	ITLGGDGTVLFASWLFQRIVPPVLSFSLGSLGFLTKFDFDHYHETLTAAFTEGVNVALRL
FG07150.1 	ISLGGDGTVLYASWLFQRIVPPVLSFALGSLGFLTKFDFEEYQETLTTAFTKGVTVSLRL
AN6824.1  	IALGGDGTVLYTSWLFQHIVPPVLSFSLGSLGFLTKFDFNDYQNILSSAIQDGVLVSLRL
          	*:********::*****:********:************:.::: *::*: .** *:***

MG08159.1 	RFEGTIMRSTRQKKVDEAGNSS--------------AASDESTSPKDERVDAEAQKKRDL
NCU03741.1	RFEGTVMRSQTNKRKLVEGDSDKKMVSNKTDKEQGGTDGPDCQDENDPSEEKEEGEKRDL
FG07150.1 	RFEGTVMRSQPRKKAQLEKGS-----------------------------DEDEEQPRDL
AN6824.1  	RFECTIMRSNPHDKETP-----------------------------------ATKKHRDL
          	*** *:***  ..:                                         : ***

MG08159.1 	VEELIGEEKDDEHTHRPDGTYEILNEVVIDRGPNPT1MSYLEIFGDDEHFTSVLADGICV
NCU03741.1	VEDLIGEEKDDERTHRPDGTWCVLNEVVVDRGPNPT1MSYIEIFGDDEHFTSVNADGICV
FG07150.1 	VEELIGEEREDEHTHRPDGTFEILNEVVVDRGPNPT1LSTTEIFGDDEHFTSVLADGICV
AN6824.1  	VDELIGDETEGTLTHRPDGVVHILNDIVVDRGPNPT1MSSIELFGGDEHFTTLQADGVCI
          	*::***:* :.  ******.  :**::*:******* :*  *:**.*****:: ***:*:

MG08159.1 	STPTGSTAYNLASGGSLCHPENPVMLVTSICPHTLSFRPIILPDTIVLRVGVPYGARTGS
NCU03741.1	STPTGSTAYNLAAGGSLCHPENPVMLVTAMCAHTLSFRPIILPDTIVLRIGVPYDARTGS
FG07150.1 	STPTGSTAYNLAAGGSLCHPENPVMLVTSICAHTLSFRPIILPDTIVLRVGVPYGARTSS
AN6824.1  	STPTGSTAYNMAAGGSLTHPDNPVILITAICAHTLSFRPIILPDTVVLRVGVPYDARTSS
          	**********:*:**** **:***:*:*::*.*************:***:****.***.*

MG08159.1 	WASFDGRERIELHPGDYVTISASRYPFASVQAPGRRSEDWVNSISAKLGWNTRQKQKAFS
NCU03741.1	WASFDGRERIELRPGDYVTISASRYPFASVQPQGRRSEEWIKSINAKLGWNTRQKQKSFT
FG07150.1 	WASFDGRERVELKPGDYVTISASRFPFASVQAEGRRSEDWVNSISGKLGWNTRQKQKSYK
AN6824.1  	WASFDGRQRVELLPGDYVTVSASRYPFANVLPHGGKGDDWMRSLSKTLNWNTRQRQKAMT
          	*******:*:** ******:****:***.* . * :.::*:.*:. .*.*****:**: .

MG08159.1 	EWQT
NCU03741.1	----
FG07150.1 	EWEK
AN6824.1  	----
          	
```
